# Expression and Display of Glycoengineered Antibodies and Antibody Fragments with an Engineered Yeast Strain

**DOI:** 10.3390/antib10040038

**Published:** 2021-09-29

**Authors:** Anjali Shenoy, Srisaimaneesh Yalamanchili, Alexander R. Davis, Adam W. Barb

**Affiliations:** 1 Department of Biochemistry and Molecular Biology, University of Georgia, Athens, GA 30602, USA; ashenoy@uga.edu (A.S.); coolmaneeshy@gmail.com (S.Y.); aledav@uga.edu (A.R.D.); 2 Complex Carbohydrate Research Center, University of Georgia, Athens, GA 30602, USA; 3 Department of Chemistry, University of Georgia, Athens, GA 30602, USA

**Keywords:** human immunoglobulin 1 (hIgG1), human Fc gamma receptor IIIa (FcγIIIa), EndoS2, truncated *N*-glycan

## Abstract

Interactions with cell surface receptors enhance the therapeutic properties of many important antibodies, including the low-affinity Fc γ Receptors (FcγRs). These interactions require proper processing of the immunoglobulin G Fc *N*-glycan, and eliminating the *N*-glycan abolishes binding, restricting antibody production to mammalian expression platforms. Yeasts, for example, generate extensively mannosylated *N*-glycans that are unsuitable for therapeutics. However, Fc with a specifically truncated *N*-glycan still engages receptors with considerable affinity. Here we describe the creation and applications of a novel *Saccharomyces cerevisiae* strain that specifically modifies the IgG1 Fc domain with an *N*-glycan consisting of a single *N*-acetylglucosamine residue. This strain displayed glycoengineered Fc on its surface for screening yeast surface display libraries and also served as an alternative platform to produce glycoengineered Rituximab. An IgG-specific endoglycosidase (EndoS2) truncates the IgG1 Fc *N*-glycan. EndoS2 was targeted to the yeast ER using the signal peptide from the yeast protein disulfide isomerase (PDI) and a yeast ER retention signal (HDEL). Furthermore, >99% of the yeast expressed Rituximab displayed the truncated glycoform as determined by SDS-PAGE and ESI-MS analyses. Lastly, the yeast expressed Rituximab engaged the FcγRIIIa with the expected affinity (*K*_D_ = 2.0 ± 0.5 μM) and bound CD20 on Raji B cells.

## 1. Introduction

Monoclonal antibodies have revolutionized the treatment of many cancers, autoimmune diseases and infectious diseases with notable high-profile examples targeting Ebola virus and COVID-19 [1,2,3,4]. Due to the strict requirement of proper post-translational modification, commercial antibodies are predominantly expressed using mammalian cells including Chinese Hamster Ovary (CHO) cells. Mammalian expression platforms produce glycoproteins with appropriate processing including asparagine(N)-linked carbohydrates (glycans) at Asn 297 of the IgG1 heavy chain that is required for interactions with the Fc γ receptors (FcγRs). These interactions trigger antibody-dependent cell-mediated cytotoxicity (ADCC) and antibody-dependent cellular phagocytosis (ADCP) that participate in clearing diseased tissue and pathogens [5,6]. *N*-glycosylation is a co-translational modification that also affects complement activation by antibodies and half-life in the serum [7,8,9,10]. Despite superior processing capabilities, mammalian expression systems require more specialized equipment and more complex growth media.

**Figure 1:**
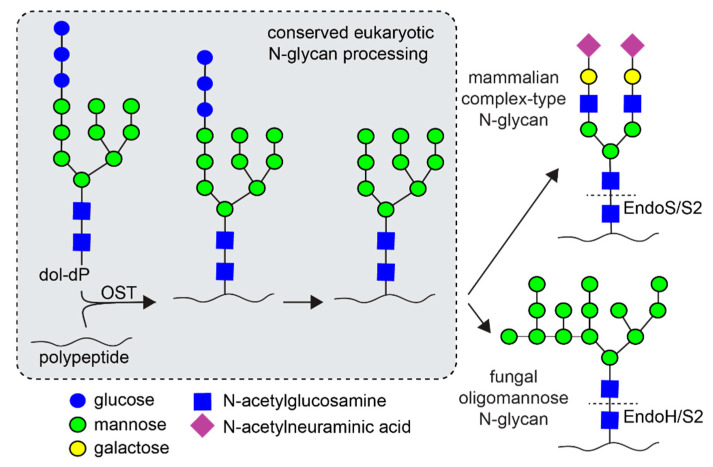
*N*-glycan processing in yeasts and mammals shares a common starting point. However, the end products are substantially different with yeasts capable of extensive mannosylation. The dashed line indicates the site of endoglycosidase (endo) cleavage; EndoS and EndoS2 cleave IgG1 *N*-glycans. OST—oligosaccharyltransferase; dol-dP—dolichol diphosphate.

Alternative microbial expression platforms have the potential to surmount limitations associated with mammalian systems. Among these, yeasts provide a distinct advantage as eukaryotes capable of *N*-glycosylation as well as other eukaryotic post-translational modifications (Figure 1) [11]. Unfortunately, antibodies produced in yeast contain oligomannose *N*-glycans which are rapidly cleared in the peripheral compartment [12]. Ideal expression platforms generate antibodies with *N*-glycan compositions similar to serum IgG, which include highly processed forms with extensive modification of the non-reducing termini [13]. It is notable that commercial mammalian antibody expression systems are imperfect and can produce undesirable amounts of oligomannose glycoforms (5–15%) that cannot be completely prevented by adjusting growth conditions [14].

It is possible to produce highly homogeneous protein *N*-glycans with alternative expression systems by enzymatic remodeling. As an alternative, several groups have attempted to introduce the glycan processing machinery into engineered yeast strains, with varying levels of success [15,16,17,18,19,20] though more successful attempts are reported in *Pichia pastoris* as compared to *Saccharomyces cerevisiae* [15,20,21]. However, competing mannosyltransferases in the Golgi lead to undesirable glycoforms in glycoengineered yeast strains and lower amounts of the glycoform of interest. Alternatively, Endoglycosidase F (Endo F) and EndoH remove the majority of the yeast *N*-glycan, which can extend to hundreds of residues in contrast to the ~8–11 IgG *N*-glycan residues in human serum (Figure 1) [22,23]. These enzymes hydrolyze *N*-glycans leaving a single *N*-acetylglucosamine residue that is identical in *N*-glycans produced by both yeast and mammals. A comparable effort targeted EndoH to the ER in a plant, *Nicotiana benthamiana*, resulting in *N*-glycan cleavage with no notable growth defects, though plants appear less dependent on *N*-glycosylation than mammalian cells [24]. This aspect allowed for an approach entailing in-vivo deglycosylation of Rituximab with EndoH in *Nicotiana benthamiana* followed by the subsequent addition of a complex-type glycan using an EndoS2 transglycosylase variant [25].

Surprisingly, antibodies and antibody fragments displaying this single-residue *N*-acetylglucosamine *N*-glycan bind FcγRIIIa with only a 10-fold reduction in affinity and elicit FcγR-mediated cellular immune responses [26,27]. The immunoglobulin-specific endoglycosidases Endo S and EndoS2 provide an alternative approach to generate antibodies with homogeneous mammalian *N*-glycans [28,29]. EndoS2 cleaves *N*-glycans from IgG1 with a broader range of *N*-glycan substrates including complex, oligomannose and hybrid type *N*-glycans [30].

Here we report the development of a yeast expression system for antibody expression. Combining the protein-expressing capabilities of *S. cerevisiae* with glycan remodeling into a single cell is expected to provide a cost benefit over post-purification protein remodeling and also provides antibody expression with inexpensive medium conditions using a host organism widely used for commercial production [31]. Another advantage of utilizing *Saccharomyces cerevisiae* over *Pichia pastoris* to create this dual application strain is the library size that can be achieved is far larger and less cumbersome to create [32]. The *Saccharomyces cerevisiae* strain described here is additionally capable of displaying the IgG1 Fc fragment on the surface, a required component of protein engineering using a yeast surface display [33].

## 2. Materials and Methods

### 2.1. Material

All materials were purchased from Millipore Sigma unless otherwise noted. GFP-FcγRIIIa was prepared as previously described [34].

### 2.2. Strain and Media

The *S. cerevisiae* strain EBY100 (GAL1-AGA1:URA3 ura3-52 trp1 leu2Δ1 his3 Δ200 pep4: HIS2 prb1 Δ1.6R can1 GAL) was purchased from the American Type Culture Collection (ATCC-MYA4941). EBY100 cells were grown in YPD rich medium. EBY100-EndoS2 yeasts were grown in minimal medium supplemented with tryptophan (Trp; YNB-Dropout + Trp = 6.7 g/L Yeast Nitrogen Base Without Amino Acids and 1.5 g/L Yeast Synthetic Drop Out Medium Supplements without Uracil, Tryptophan and Leucine along with 2 mg/mL L-Trp). EBY100-EndoS2 + pYD1-Fc yeast cells were grown in a minimal medium lacking leucine and tryptophan (YNB-Dropout = 0.67 g/L Yeast Nitrogen Base Without Amino Acids and 0.15 g/L Yeast Synthetic Drop Out Medium Supplements without Uracil, Tryptophan and Leucine). Protein expression was induced in YPGal = 10 g/L Yeast Extract + 20 g/L Peptone + 2% Galactose (*w/v*).

### 2.3. Cloning of Fc into the Yeast Surface Display Plasmid pYD1

pYD1 was purchased from Addgene. A codon optimized Fc insert for expression in *S. cerevisiae* was ordered from IDT. This insert was cloned into the pYD1 plasmid using restriction sites Nhe1 and EcoR1. The final plasmid pYD1-Fc was validated through DNA sequencing (Eurofins).

### 2.4. Creation and Validation of the EBY100-EndoS2 Strains

The construct encoding the app8 signal peptide with EndoS2, a Flag tag followed by the HDEL sequence was synthesized by IDT, then cloned into the Yeast Integrating Plasmid (YIP) pAG305 (Addgene) to integrate EndoS2 into the LEU2 site in the yeast genome. Once the sequence was validated through DNA Sequencing, the plasmid was linearized and used to transform EBY100. The integration was validated through yeast colony PCR. The positive cells were then transformed with the pYD1-IgG1 Fc yeast surface display plasmid. This transformation was again validated through a PCR-based colony screen using primers listed in Table S1. To determine whether the endogenous EndoS2 can cleave the *N*-glycan off Fc, the cells were first grown in YNB-Dropout medium [6.7 g/L Yeast Nitrogen Base without amino acids and ammonium sulfate (Sigma Y1251) and 1.5 g/L Yeast Synthetic Dropout Medium Supplement (Sigma Y1771)] supplemented with 2% glucose and then induced in YNB-Dropout supplemented with 2% galactose for 24 h. The OD_600_ of the culture was measured and a volume of culture corresponding to ~2 × 10^8^ colony-forming units was taken to prepare protein samples for a Western blot (0.1 OD_600_~10^6^ cells).

### 2.5. Protein Sample Preparation

Protein samples for Western blots were prepared by concentrating cells with centrifugation for 2 min at 1500× *g*. The pellet was then washed three times with 100 µL of 1× PBS, followed by an incubation in 100 µL of 0.1 M NaOH and incubation at RT for 5 min. The cells were then spun down at 1500 g for 2 min, then resuspended in 50 µL of 2× SDS PAGE sample buffer. The cells were incubated at 95 °C for 5 min. The sample was then centrifuged for 10 min at 1500× *g*. Supernatant (10 µL) was diluted 1:1 using 2× SDS PAGE sample buffer and was loaded onto a 12% SDS polyacrylamide gel. The protein was transferred to a polyvinylidene difluoride (PVDF) membrane using the Invitrogen Power Blotter (PB0010). The membrane was blocked in TBS + Tween20 (TBST) with 5% dry milk for one hour at room temperature on an orbital shaker. This was followed by incubation with rabbit anti-hIgG primary antibody (Target: IgG; Rabbit IgG; AB_228410 ThermoFisher) in 5% dry milk in 1× TBST overnight at 4 °C on an orbital shaker. The membrane was washed three times for 5 min with TBST buffer, followed by incubation with secondary HRP conjugated antirabbit antibody (Target: Rabbit IgG Clone: Polyclonal Goat IgG; Research and Diagnostic Systems, dilution 1:2000) in 5% milk in 1× TBST buffer for 2 h at room temperature on an orbital shaker. The membrane was washed three times with 1× TBST buffer for 5 min and then imaged using ChemiDoc XRS + Image System (Bio-Rad, Hercules, CA, USA) using the ECL Western Blotting Substrate (Thermo Fisher Scientific, Waltham, MA, USA).

### 2.6. Flow Cytometry to Determine Fc Surface Expression

EBY100-EndoS2 + pYD1-Fc cells were first grown in YNB-Dropout + 2% glucose overnight, pelleted by centrifugation and then induced in YNB-Dropout + 2% galactose for 24 h at 30 °C with shaking. Cells (~10^6^) from this culture were used to prepare samples for flow cytometry. The pellet was washed with 100 µL of 1× PBS and then washed with 100 µL of 1× PBSA (1× PBS + 1% Bovine Serum Albumin). The cells were incubated with rabbit anti-hIgG on ice for 45–60 min in the dark. The cells were then washed with 100 µL of 1× PBSA. The cell pellet was then incubated with phycoerythrin-conjugated antirabbit IgG on ice for 45–60 min in the dark. The cell pellet was then washed twice with 100 µL 1× PBS. Similarly, for GFP-FcγRIIIa staining, a cell pellet ~10^6^ cells were incubated with 50 μL of ~2 mM GFP-FcγRIIIa for 1 h at 4 °C in the dark. The samples were analyzed with a FACSCanto to determine Fc surface expression and receptor FcγRIIIa binding.

### 2.7. Expression and Purification of IgG1 Fc and Rituximab

A single yeast clone verified through the colony screen was inoculated into a 5 mL YNB-Dropout medium supplemented with 2% glucose and incubated with shaking at 30 °C for 16 h. This culture was then added to 100 mL YNB-Dropout supplemented with 2% galactose, then incubated with shaking for 24 h at 30 °C. The culture was then centrifuged for 10 min at 3000× *g*. The supernatant was discarded. The cell pellet was then resuspended with 100 or 500 mL YPGal and was incubated with shaking for 72 h at 20 °C. This culture was then centrifuged twice at 3000× *g* for 10 min to remove cell debris. The supernatant was diluted 2-fold with 25 mM MOPS 100 mM NaCl, pH 7.4 (Buffer A) and then loaded onto a Protein A column (2 mL). The column was then washed with 12 mL of Buffer A. Protein was eluted in 1 mL fractions of 100 mM glycine, pH 3.0 and was collected in tubes containing 500 μL of 1 M Tris pH 8 to immediately neutralize it. The protein samples were then loaded onto a 12% SDS-PAGE gel to be analyzed. For samples analyzed with size exclusion chromatography: 8–10 mL of concentrated sample was applied to a Superdex 200 column using an ÄKTA Go FPLC (Cytiva) prewashed with Buffer A and eluted with Buffer A.

### 2.8. Purification Using Concanavalin A Column

Concanavalin A Sepharose resin (1 mL) was washed with 5 column volumes of Equilibration Buffer. Purified IgG1 Fc was diluted 1:1 with Equilibration Buffer (1× PBS pH 7.4, 0.1 M NaCl, 1 mM MnCl_2_ and 1 mM CaCl_2_), then loaded onto the column by gravity. The column was then washed twice using 5 mL of Equilibration Buffer. The protein was then eluted from the column as 1 mL fractions using Elution Buffer (1× PBS pH 7.4, 0.2 M d-mannose). Each fraction (10 µL) was analyzed using a 12% SDS-polyacrylamide gel that was stained with Coomassie brilliant blue.

### 2.9. Cloning the Heavy Chain and Light Chain of Rituximab into pESC-TRP1

The constructs for the app8-heavy chain and app8-light chain were synthesized by IDT. These constructs were then inserted into pESC-TRP1 (Agilent) through restriction endonuclease cloning using Sal1 and Nhe1 (heavy chain) and EcoRI and Nhe1 (light chain) and ligated with T4 DNA Ligase according to the manufacturer’s directions. The completed sequence was validated through DNA sequencing (Eurofins).

### 2.10. Mass Spectrometry

Purified Rituximab (5 µg) from the EBY100-(GPD) EndoS2 strain was resuspended in 50 mM ammonium carbonate pH 8.0, 10% methanol, boiled for 5 min at 95 °C, then cooled on ice for 5 min. The sample was then treated with 5 mM dithiothreitol and incubated at 37 °C for 1 h, then 14 mM iodoacetamide was added and the sample incubated in the dark for 30 min at room temperature before adding trypsin (~1.8 μg) and incubating at 37 °C overnight. The samples were then lyophilized. The samples were then resuspended in binding buffer (15 mM ammonium acetate, 85% acetonitrile, pH 3.5). This solution was loaded onto a Cellulose/HILIC column, which was then washed three times with 10 μL of binding solution. Glycopeptides were eluted with 15 mM ammonium acetate, 10% acetonitrile, pH 3.5 to collect three elution fractions (10 μL each).

### 2.11. Surface Plasmon Resonance

Rituximab was immobilized on a CM5 chip (Cytiva) as previously described in [35] using a BiaCore T200 instrument (Cytiva). Increasing concentrations of GFP-FcγRIIIa were applied to the chip surface and each concentration was continuously applied until equilibrium was reached. Affinity constants were calculated using the T200 system software after double normalization (first to the surface-deactivated reference channel and then to a sample with zero concentration collected using the lane of interest) and a 1:1 binding model with time zero = zero response units. Data were averaged from a 5 s window after equilibrium was reached. Reported errors indicate errors of the curve fitting procedure.

### 2.12. Flow Cytometry for Fab Functionality with Raji B-Cells

Raji B-cells (5 × 10^5^) were washed with 1× PBS. Antibody dilutions were prepared in 1× PBS + 1% FBS. The cells were spun down at 400 g for 5 min. The cells were blocked with 90 μL 1× PBS + 1% FBS + 10 μL human serum and incubated for 5 min on ice. Antibody dilutions of the yeast-derived Rituximab and commercial Rituximab were added to the cells as separate samples. The cells were incubated on ice for 1 h. The cells were centrifuged at 400× *g* for 5 min and resuspended in 100 μL of 1× PBS twice. This was followed by another blocking step with human serum described above and the addition of 1 μg per sample of rabbit anti-hIgG1 antibody (Target: Human IgG; Clone: Rabbit; RRID: AB_228410). This incubation was carried out on ice for 30 min. The cells were centrifuged at 400× *g* for 5 min twice and resuspended in 100 μL of 1× PBS twice. This was followed by another blocking step with human serum described above followed by the addition of a secondary PE-conjugated donkey antirabbit antibody (Target: Human IgG; Clone: Donkey: RRID: AB_2563484). This incubation was carried out on ice for 30 min. The cells were centrifuged at 400× *g* for 5 min and resuspended in 100 μL of 1× PBS twice. The samples were then blocked with 1× PBS + 1% paraformaldehyde for 20 min at RT. The samples were then run on the CytoFLEX and analyzed.

## 3. Results

### 3.1. IgG1 Fc with a Hydrolyzed N-Glycan Expressed on Yeast Binds GFP-FcγRIIIa

We integrated *endoH* into *S. cerevisiae* (EBY100) and fused DNA encoding a signal peptide from the yeast protein disulfide isomerase (PDI) gene and a yeast ER retention signal (HDEL) [36]. We selected the HO locus because integration at this site occurs with high efficiency and negligible impact on cell growth [37]. EndoH expression in this strain is controlled by the GAL1 promoter to minimize deleterious effects on cell growth. Unfortunately, this strain exhibited growth defects and inefficient *N*-glycan cleavage (Supplemental Figure S1). It is possible that because EndoH cleaves all *N*-glycosylated yeast proteins, growth was inhibited by off-target cleavage of glycans on proteins that are important for proper cell function, therefore we pursued incorporating an IgG-specific *N*-glycan hydrolase.

**Figure 2:**
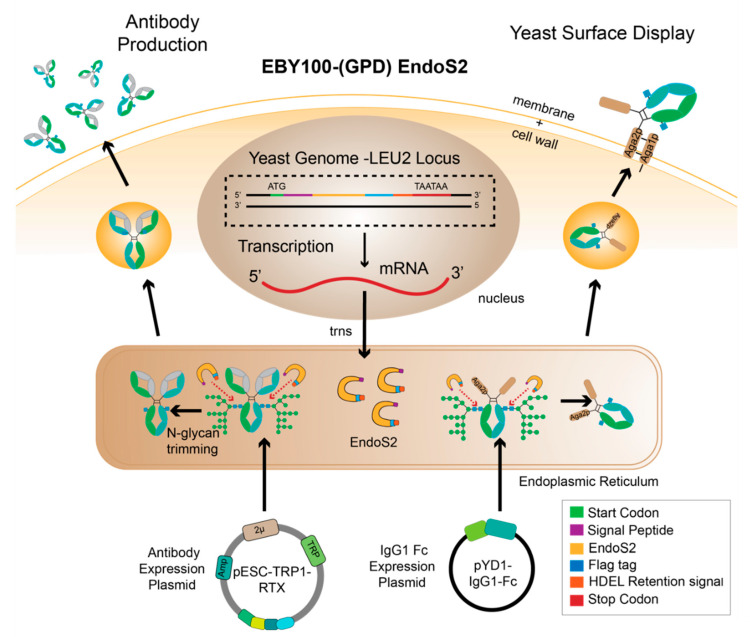
EBY100-(GPD) EndoS2 is a dual-function yeast strain that can display glycoengineered Fc on the yeast surface for protein engineering or produce glycoengineered antibodies.

**Figure 3:**
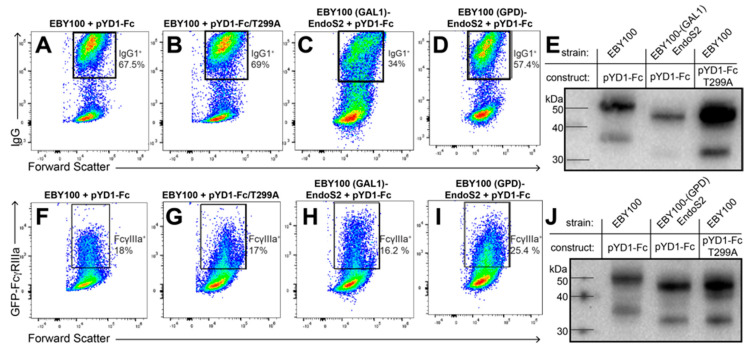
IgG1 Fc display and processing by the EndoS2 yeast strains and the respective controls (pYD1-Fc and pYD1-Fc/T299A). Flow cytometry for IgG1 Fc using an anti-IgG1 antibody (**A**–**D**) and GFP-FcγRIIIa binding (**F**–**I**). Boundaries for the highlighted boxes were drawn based on negative controls (not shown). (**E**,**J**) Western blots showing mobility of the Aga2p-IgG1 Fc fusions. T299A is an aglycosylated IgG1 Fc variant which shows reduced binding to GFP-FcγRIIIa.

We integrated *endoS2* into the yeast LEU2 locus [38]. This inserted construct likewise targeted EndoS2 to the ER and included an ER retention tag, as described for EndoH, above (Figure 2). We observed a shift in mobility of yeast surface displayed IgG1 Fc expressed in the EBY100-(GAL1) EndoS2 comparable to the aglycosylated version of the same protein (Figure 3E). This result suggests that endogenously produced EndoS2 efficiently cleaved the Fc *N*-glycan. However, this strain showed low surface expression of IgG1 Fc and low GFP-FcγRIIIa binding (Figure 3C,H). It is possible that utilizing the GAL1 promoter for expressing both the EndoS2 and IgG1 Fc resulted in lowered expression of both proteins.

To overcome reduced expression, we next incorporated *endoS2* with a constitutive GPD promoter into the EBY100 strain and continued to express IgG1 Fc from pYD1 with the GAL1 promoter. These promoters reportedly permitted high levels of simultaneous expression [29]. The EBY100-(GPD) EndoS2 strain resulted in superior Fc surface expression and GFP-FcγRIIIa surface staining (Figure 3D,I). Furthermore, we observed efficient *N*-glycan cleavage (Figure 3J).

### 3.2. IgG1 Fc with a Truncated N-Glycan Is Secreted by EBY100-(GPD) EndoS2

We attempted to extract IgG1 Fc to further validate the glycoform produced by the EBY100-(GPD) EndoS2 strain. The Aga2p-Fc expressed from the pYD1-Fc plasmid is anchored onto the yeast surface through disulfide bonds with the yeast cell wall protein Aga1p [33]. We failed to break these bonds using dithiothreitol and recover a sufficient amount of sample for analysis [39,40]. Furthermore, the Aga2p-fused IgG1 Fc appears as two bands in SDS-PAGE with the upper band likely resulting from Aga2p O-glycosylation (Figure 3C,F). As an alternative, we prepared a plasmid to secrete IgG1 Fc into the medium by appending the engineered *N*-terminal app8 secretion factor in place of the Aga2p fusion [41].

**Figure 4:**
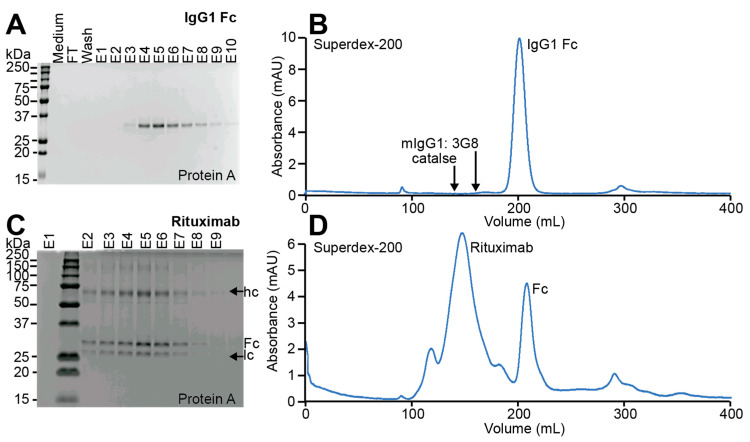
Purification of IgG1 Fc and Rituximab expressed from EBY100-(GPD)EndoS2. IgG1 Fc purified using Protein A chromatography (**A**) and size-exclusion chromatography (**B**). Rituximab purified using Protein A chromatography (**C**) and size-exclusion chromatography (**D**). The largest peak in D at 140 mL is Rituximab. Arrows indicate the Rituximab heavy and light chain. (**A**,**C**) are 12% reducing SDS-PAGE gels.

We purified IgG1 Fc secreted by both the EBY100 and EBY100-(GPD) EndoS2 strain from the spent culture medium using Protein A chromatography [42]. Protein A, a cell wall component of *Streptococcus aureus*, binds the IgG1 Fc domain [43]. The IgG1 Fc proteins isolated from both yeast strains bound Protein A and were eluted using 100 mM glycine, pH 3.0 (the single *N*-acetylglucosamine form is shown in Figure 4A and Figure S2).

**Figure 5:**
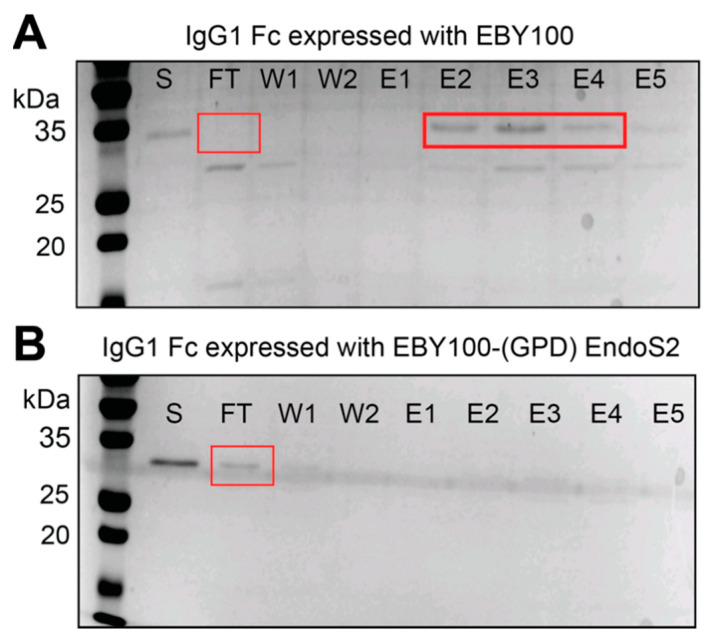
Identification of oligomannose *N*-glycans on secreted IgG Fc. Concanavalin A purification of Fc derived from the (**A**) EBY100 and the (**B**) EBY100-(GPD) EndoS2 strains. “S” is the starting material before loading on the column, “FT” is the flow-through unbound fraction, “W” wash and “E” elutions.

We next probed the secreted IgG1 Fc for the presence of oligomannose-type *N*-glycans using a concanavalin A-sepharose resin. IgG1 Fc isolated from wildtype EBY100 cells is expected to possess an oligomannose glycoform, and we observed binding of the majority of this protein to the concanavalin A column (Figure 5A). A small amount of Coomassie stained material is observed at a substantially lower molecular weight in the flow-through fraction. This may represent a contaminating protein in the preparation or aglycosylated Fc. Furthermore, we observed that the majority of the IgG1 Fc isolated from our glycoengineered EBY100-(GPD) EndoS2 strain did not bind the concanavalin A column, which is consistent with the EndoS2-catalyzed removal of the *N*-glycan leaving a single *N*-acetylglucosamine residue (Figure 5B).

### 3.3. Glycoengineered Rituximab Expressed with EBY100-(GPD) EndoS2

We next determined whether the EBY100-(GPD) EndoS2 strain would produce a full-length glycoengineered antibody and serve as an alternative antibody expression platform. Wong and coworkers created glycoengineered antibodies with glycoengineered *Pichia pastoris* and utilized a bidirectional promoter to produce both heavy chain and light chain from a single construct [44]. We adopted a similar approach using the pESC-TRP1 plasmid with a bidirectional GAL1-GAL10 promoter to express Rituximab (Figure 2). We chose to use the GAL1 promoter for expressing the heavy chain based on the reported stronger expression compared to GAL10 [45]. Similar to the IgG1 Fc expression construct, we appended the app8 secretion factor at the *N*-termini of both the heavy chain and light chain. The pESC-TRP1-app8RTXhc-app8RTXlc was transformed into the EBY100-(GPD) EndoS2 strain. A positive colony was then grown and induced to produce full-length Rituximab.

### 3.4. Purification of Full-Length Rituximab from the EBY100-(GPD) EndoS2 Strain

We purified full-length Rituximab from the spent medium of an induced yeast culture using a Protein A column. Rituximab isolated from the EBY100-(GPD) EndoS2 strain bound protein A, signifying that the antibody was properly folded, and eluted with 100 mM glycine, pH 3.0, as is expected for IgG1 (Figure 4C).

Yeast-expressed Rituximab eluted from a Superdex 200 column at 140 mL as shown in Figure 4D. This retention time is between the peaks for catalase (206 kDa) and 3G8 (a mouse IgG1; 145 kDa, Figure 4B,D) and is consistent with a heterotetrameric assembly containing two heavy chains and two light chains with an expected molecular mass of 145 kDa. Additionally, we noted the presence of IgG1 Fc in the purified sample that is likely the result of proteolysis during Rituximab expression and retained during the protein A separation step (Figure 4D). We also observed some minor peaks in Rituximab likely resulting from aggregation and proteolysis (Figure 4D). We recovered 0.24 mg of purified Rituximab from a 500 mL culture at a yield of 0.48 mg/L, which was more than twice the amount of Rituximab we were able to recover from our unmodified yeast strain (0.2 mg/L).

**Figure 6:**
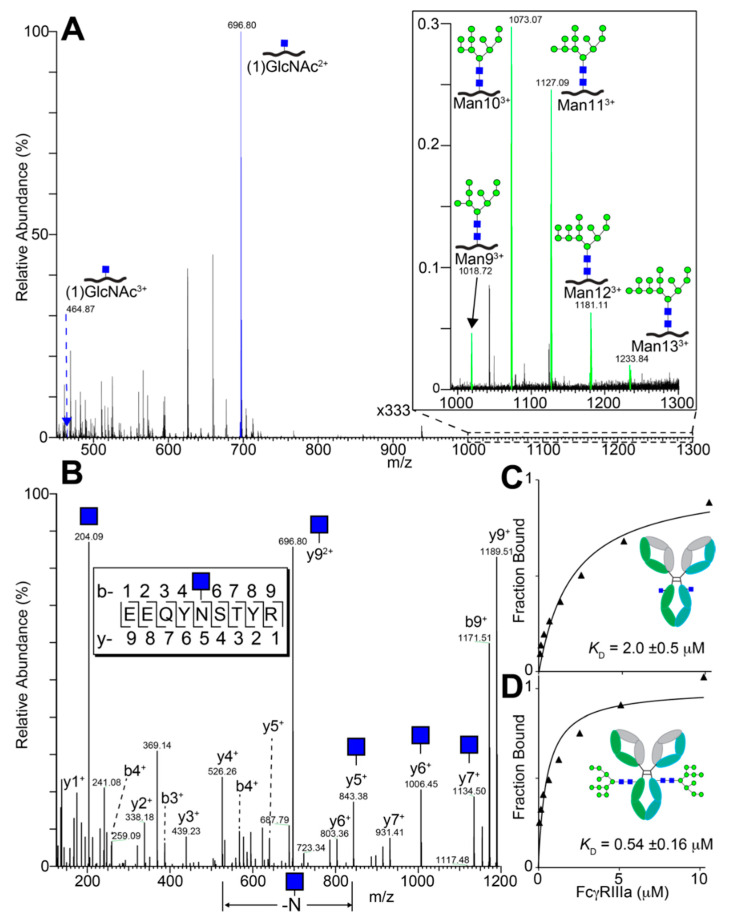
Yeast-expressed Rituximab composition and receptor binding. (**A**) Total ion current from ESI-MS spectra of trypsinized Rituximab expressed from the EBY100-(GPD) EndoS2 strain summed from 11–15 min; doubly charged ions corresponding to oligomannose *N*-glycoforms were not observed. Isobaric ions were not distinguished, thus each cartoon represents multiple possible configurations. (**B**) ESI-MS/MS of the (1) GlcNAc glycopeptide from panel A showing a single GlcNAc residue at N297. Rituximab displaying either truncated (**C**) or yeast *N*-glycans (**D**) binding to high affinity FcγRIIIa (V158) variant as determined by surface plasmon resonance.

We characterized the purified yeast-expressed Rituximab by electrospray ionization tandem mass spectrometry (Figure 6). The most predominant glycopeptide peak belonged to the N297-containing tryptic peptide modified with a single *N*-acetylhexosamine residue. An MS2 spectrum of this species identified individual peaks following collision-induced degradation, including the loss of a single *N*-acetylhexosamine residue coincident with the loss of N297, indicating N297 as the likely site of the carbohydrate addition (Figure 6B). Based on comparing the relative intensity of MS1 peaks for the N297-containing species, glycopeptides with a single *N*-acetylglucosamine (99.36%) appeared as the most abundant form with lesser amounts of Man_9_ (0.04%), Man_10_ (0.29%), Man_11_ (0.24%), Man_12_ (0.06%) and Man_13_ (0.01%; Figure 6A). Furthermore, we identified peptides covering 93.9 and 100% of the heavy and light chain sequences, respectively, and found no spectra corresponding to the app8 secretion tag on the light chain and 24 app8 peptides out of 1845 total for the heavy chain, indicating a high level of app8 cleavage during antibody secretion (Supplemental MSExcel worksheet). The high rate of EndoS2-catalyzed *N*-glycan cleavage determined by MS is consistent with the high level of IgG1 Fc processing observed in SDS-PAGE and concanavalin A binding (Figure 3 and Figure 5). These results demonstrate that the endogenous EndoS2 efficiently cleaved the *N*-glycan from Rituximab and IgG1 Fc.

### 3.5. Functionality of Rituximab Isolated from EBY100-(GPD) EndoS2

We next assessed the functionality of the EBY100-(GPD) EndoS2 expressed Rituximab. First, we analyzed binding to GFP-FcγRIIIa using surface plasmon resonance (SPR) by immobilizing Rituximab to a chip and flowing over various amounts of GFP-FcγRIIIa (Figure 6C). We determined a *K*_D_ of 2.0 ± 0.5 μM which was slightly lower than the 5.8 μM value previously reported for an identical glycoform of IgG1 Fc [27]. Rituximab with the non-truncated yeast *N*-glycan shows ~4-fold greater affinity (Figure 6D), which is identical to previous measurements of IgG1 Fc with an oligomannose *N*-glycan [27].

**Figure 7:**
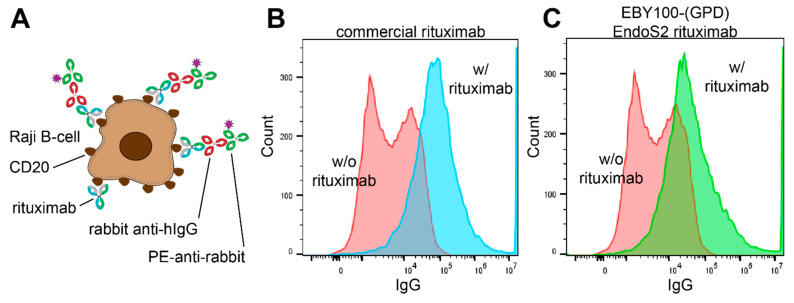
Binding of yeast-expressed Rituximab to Raji B cells. (**A**) Schematic diagram of the detection strategy. Flow cytometry analysis of Raji B cells incubated with the two detection antibodies shown in panel A, with or without commercially sourced Rituximab (**B**) or EBY100-(GPD) EndoS2 expressed Rituximab(**C**).

We next tested the ability of the EBY100-(GPD) EndoS2 expressed anti-CD20 Rituximab to bind CD20-expressing Raji B-cells by incubating this antibody with Raji B-cells and detecting the amount of Rituximab on the cell surface using flow cytometry. We saw a clear increase in anti-IgG staining following this treatment that was comparable to a commercial Rituximab preparation, indicating both Rituximab antibodies efficiently bound Raji B cells a ta concentration of 40 nM (Figure 7). Furthermore, we observed the same amount of staining for both the commercial and yeast derived Rituximab at 400 nM (Supplemental Figure S3) [46].

## 4. Discussion

The IgG1 Fc *N*-glycan plays a crucial role in the interaction between IgG1 and FcγRIIIa. The IgG1 Fc *N*-glycan does not directly engage the receptor; however, it has been shown to stabilize the C’E loop that interacts with the receptor [27]. The IgG1 Fc *N*-glycan also forms contacts with aromatic amino acids on the Fc domain through CH-π bonds, providing further stabilization [27]. The composition of the *N*-glycan has also been shown to influence FcγRIIIa binding affinity [47,48]. Removal of this *N*-glycan abolishes receptor binding; however, our group demonstrated that trimming the Fc *N*-glycan to a single *N*-acetylglucosamine residue still allows for receptor engagement (10-fold lower affinity compared to wildtype interaction) [27]. The efficacy of this truncated glycoform has likewise been shown through cell-based assays [49]. Therefore, we sought to create a yeast strain that could produce antibodies with the single *N*-acetylglucosamine glycoform to achieve the two goals of (i) preserving FcγRIIIa binding and (ii) removing the oligomannose glycoforms that promote clearance.

One approach to creating this strain would be to knockout ER and Golgi resident mannosyltransferases to trim down the *N*-glycan. However, such knockout strains have shown defects in morphology and several growth defects that are expected to reduce commercial viability [50]. Therefore, we pursued a targeted approach that would allow us to specifically trim the IgG1 Fc *N*-glycan without causing growth defects in yeast. We achieved this through the creation of the EBY100-(GPD) EndoS2 strain. In this strain, the use of EndoS2 specifically cleaves the *N*-glycan from Fc and eliminates off-target effects. We observed a high level of *N*-glycan processing (>90%) in Rituximab produced by this strain through mass spectrometry experiments. This result surmounts previous efforts that report substantial difficulty glycoengineering *S. cerevisiae* [21].

The EBY100-(GPD) EndoS2 strain also proved effective to express full-length functional Rituximab. Previously, full-length antibodies were produced in *S. cerevisiae* at very low levels of 50 μg/L [51]. Work performed by Wittrup and co-workers lead to the development of the app8 secretion tag that has allowed for enhancement in antibody production [41]. Utilizing this secretion tag, we were able to recover ~500 μg/L of highly processed Rituximab with a high level of processing and complete removal of the app8 secretion tag. Though it is unlikely that this yeast platform will affect commercial therapeutic antibody manufacture that utilizes highly optimized CHO cells and boasts yields > 2 g/L, there is substantial potential to improve yield in this strain with further strain engineering and promote laboratory-scale antibody development efforts [11,52].

Antibodies expressed with the EBY100-(GPD) EndoS2 strain are likewise ideal substrates for post-purification glycan remodeling. Novel transglycosylase EndoS and EndoS2 variants are available for modification of antibodies and require antibodies with an *N*-glycan consisting of only a single *N*-acetylglucosamine residue and a synthetic sugar donor [53,54]. These reactions boast a high level of efficiency [55]. One antibody *N*-glycan remodeling approach entailed a combination of glycoengineered yeast strains and chemoenzymatic reactions to engineer Herceptin with a complex-type *N*-glycoform. This method required producing Herceptin in knock-out *Pichia pastoris* strains, followed by the removal of the *N*-glycan using an endoglycosidase and then adding the *N*-glycan back using transglycosylase EndoS2 variants [44]. The EBY100-(GPD) EndoS2 strain can be used to replace separate expression and in vitro truncation steps and reduce cost considerably due to its high efficiency of *N*-glycan cleavage. The antibody produced by the EBY100-(GDP) EndoS2 strain can then be remodeled following purification to have the desired glycoform using any of these transglycosylase enzyme variants.

In addition to antibody expression, the EBY100-(GPD) EndoS2 strain displays glycoengineered Fc on the yeast surface, can be used to screen yeast surface display libraries, and offers an inexpensive alternative platform to produce glycoengineered antibodies.

In summary, we report the development and characterization of a novel *S. cerevisiae* that efficiently expresses antibodies and antibody fragments that contain a truncated N297 glycan.

## Supplementary Materials

The following are available online at https://www.mdpi.com/article/10.3390/antib10040038/s1, Figure S1: Western blot showing Aga2p-Fc fusion mobility in the EBY100-(GAL1)-Endo H strain compared to an aglycosylated control (T299A), Figure S2: Fc purification using Protein A starting with protein expressed from (A) the unmodified yeast strain EBY100 and (B) the glycoengineered yeast strain EBY100-(GPD)-EndoS2. Smearing in E2 in panel A likely represents extensive *N*-glycan processing, Figure S3: Rituximab binding to Raji B cells (A) 400 nM commercial Rituximab (B) 400 nM Rituximab from the EBY100-(GPD) EndoS2 strain, Table S1: List of primers used for strain creation.
