# Supplementary material for: Expression and Display of Glycoengineered Antibodies and Antibody Fragments with an Engineered Yeast Strain
Source: Antibodies (Basel). 2021 Sep 29;10(4):38. doi: 10.3390/antib10040038 (PMC8544235; doi:10.3390/antib10040038)
Supplement: Supplementary file 1 [file antibodies-10-00038-s001.zip › Supplement_092121.pdf]

Supplement for

Expression and display of glycoengineered antibodies and antibody fragments with an engineered yeast strain

Anjali Shenoy[1], Srisaimaneesh Yalamanchili[1], Alexander R. Davis[1] and Adam W. Barb[1,2,3,*]

[1]Department of Biochemistry and Molecular Biology
[2]Complex Carbohydrate Research Center
[3]Department of Chemistry
University of Georgia, Athens, GA
*   Correspondence: author email: abarb@uga.edu

Table S1. List of primers used for strain creation.

Primer Name	Sequence	Purpose
ESFor	5'GCC GCT CTA GAA TGAAGTTTTCTG '3	Amplification Forward primer
ESRev	5'GCCGGACC TAA GCT TCT ATT AAAG '3	Amplification Reverse primer
ESSeq_F	5' GTT TTC TGC TGG TGC CGT C '3	Sequencing forward primer
ESSeq_R	5' GCC AAG CTC GGA ATT AAC CCT C '3	Sequencing reverse primer
ESYC_F	5' GCA TCT TAC GAT ACC TGA GTA TTC CC	Yeast Colony PCR – EndoS2 insert- Forward
ESYC_R	5' CAA GAC AGG ACG GCA CCA GC '3	Yeast colony PCR-EndoS2- Reverse primer
YCol-F	5' GTCAGTAATTGCGGTTCTCAC '3	Yeast Colony PCR – pYD1 insert – Forward
YCol-R	5' GCCTTTCGCTTTGGAAATTGTC '3	Yeast Colony PCR – pYD1 insert -Reverse
HCFor	CTAGCGTCGACATGAGGTTCCCGTC	Amplify app8 for fusion PCR with Heavy
AFHCRev	CTGTAATTGAACTTGGGCTTCTGCTTCTC	Amplify app8 for fusion PCR with Heavy
AFHCFor	GAG AAG CAG AAG CCC AAG TTC AAT TAC AG	Amplify Heavy chain for fusion PCR with app8
HCR	CGT AGC GCT AGC TTA TTA CTT TCC CGG	Amplify Heavy chain for fusion PCR with app8
LCFor	GTT GAA TTC ATG AGG TTC CCG TCT ATT TTT ACG	Amplify app8 for fusion PCR with Light Chain
AFLCRev	GAACAATCTGGGCTTCTGCTTCTCTC	Amplify app8 for fusion PCR with Light Chain
AFLCFor	GAG AGA AGC AGA AGC CCA GAT TGT TC	Amplify Light Chain for fusion PCR with app8
LCR	GATCTGCGGCCGCTTATCAACATTCTC	Amplify Light Chain for fusion PCR with app8
GAL1	5' ATTTTCGGTTTGTATTACTTC 3'	DNA Sequencing Forward Primer (From
GALI reverse	5' GTTCTTAATACTAACATAACT 3'	DNA Sequencing Reverse Primer (From
GALI0	5' GGTGGTAATGCCATGTAATATG 3'	DNA Sequencing Forward Primer (From
GALI0	5' GGCAAGGTAGACAAGCCGACAAC 3'	DNA Sequencing Reverse Primer (From

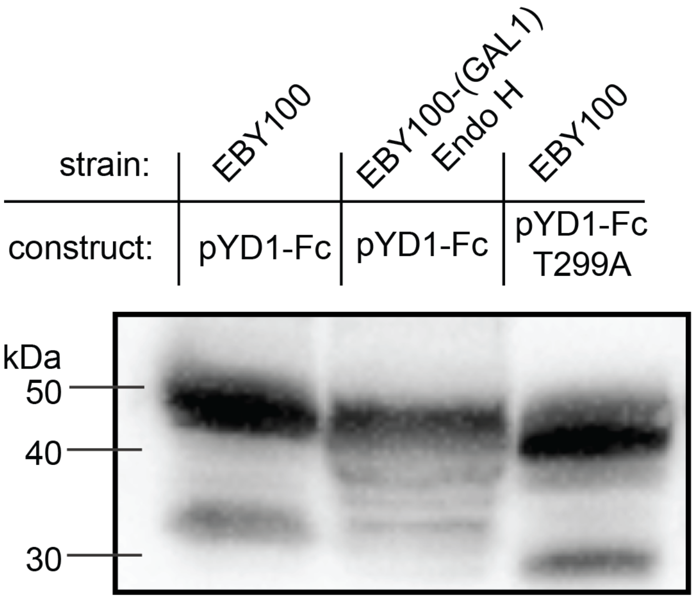

Supplementary Figure S1. Western blot showing Aga2p-Fc fusion mobility in the EBY100-(GAL1)-Endo H strain compared to an aglycosylated control (T299A).

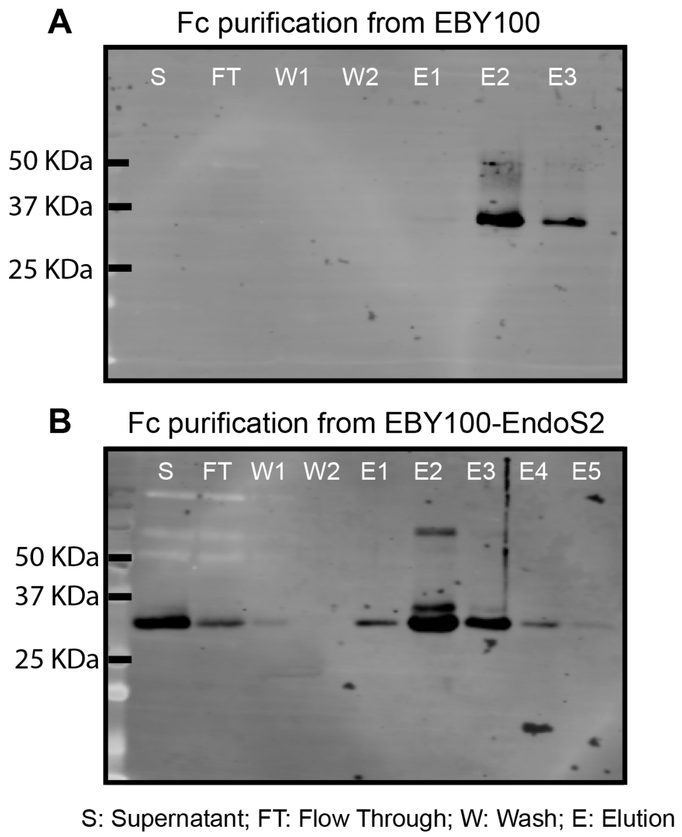

Supplementary Figure S2. Fc purification using Protein A starting with protein expressed from (A) the unmodified yeast strain EBY100 and (B) the glycoengineered yeast strain EBY100-(GPD)-EndoS2. Smearing in E2 in panel A likely represents extensive N-glycan processing.

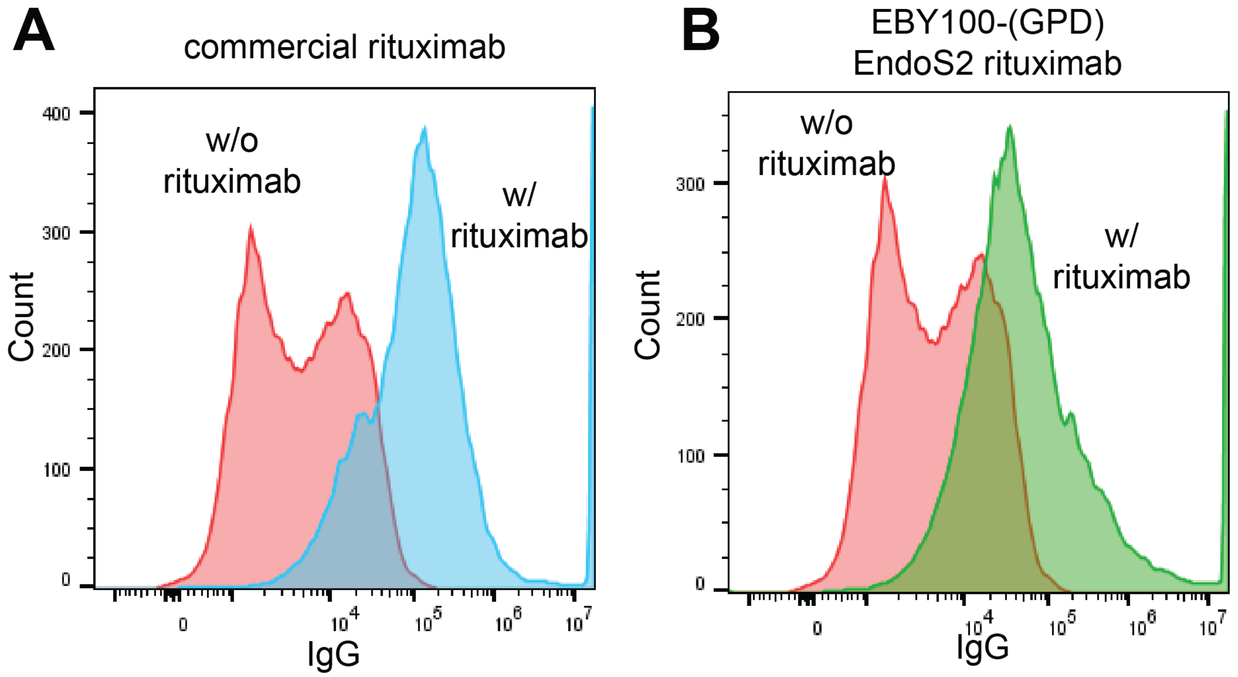

Supplementary Figure S3. Rituximab binding to Raji B cells (A) 400 nM commercial Rituximab (B) 400 nM Rituximab from the EBY100-(GPD) EndoS2 strain.

Protein Sequences:

EndoS2 Protein Sequence [Signal Peptide – EndoS2 – Flag tag- HDEL]

MKFSAGAVLSWSSLLLASSVFAQQEAEEKTVQTGKTDQQVGAKLVQEIREGKRGPLYA
GYFRTWHDRASTGIDGKQQHPENTMAEVPKEVDILFVFHDHTASDSPFWSELKDSYVH
KLHQQGTALVQTIGVNELNGRTGLSKDYPDTPEGNKALAAAIVKAFVTDRGVDGLDIDI
EHEFTNKRTPEEDARALNVFKEIAQLIGKNGSDKSKLLIMDTTLSVENNPIFKGIAEDLDY
LLRQYYGSQGGEAEVDTINSDWNQYQNYIDASQFMIGFSFFEESASKGNLWFDVNEYD
PNNPEKGKDIEGTRAKKYAEWQPSTGGLKAGIFSYAIDRDGVAHVPSTYKNRTSTNLQR
HEVDNISHTDYTVSRNLKTLMTEDKRYDVIDQKDIPDPALREQIIQQVGQYKGDLERYN
KTLVLTGDKIQNLKGLEKLSKLQKLELRQLSNVKEITPELLPESMKKDAELVMVGMTGL
EKLNLSGLNRQTLDGIDVNSITHLTSFDISHNSLDLSEKSEDRKLLMTLMEQVSNHQKIT
VKNTAFENQKPKGYYPQTYDTKEGHYDVDNAEHDILTDFVFGTVTKRNTFIGDEEAFAI
YKEGAVDGRQYVSKDYTYEAFRKDYKGYKVHLTASNLGETVTSKVTATTDETYLVDV
SDGEKVVHHMKLNIGSGAIMMENLAKGAKVIGTSGDFEQAKKIFDGEKSDRFFTWGQT

NWIAFDLGEINLAKEWRLFNAETNTEIKTDSSLNVAKGRLQILKDTTIDLEKMDIKNRKE
YLSNDENWTDVAQMDDAKAIFNSKLSNVLSRYWRFCVDGGASSYYPQYTELQILGQRL
SNDVANTLKDDYKDDDDKHDEL

Heavy Chain of Rituximab (Protein Sequence without app8)

QVQLQQPGAELVKPGASVKMSCKASGYTFTSYNMHWVKQTPGRGLEWIGAIYPGNGD
TSYNQKFKGKATLTADKSSSTAYMQLSSLTSEDSAVYYCARSTYYGGDWYFNVWGAG
TTVTVSAASTKGPSVFPLAPSSKSTSGGTAALGCLVKDYFPEPVTVSWNSGALTSGVHTF
PAVLQSSGLYSLSSVVTVPSSSLGTQTYICNVNHKPSNTKVDKKVEPKSCDKTHTCPPCP
APELLGGPSVFLFPPKPKDTLMISRTPEVTCVVVDVSHEDPEVKFNWYVDGVEVHNAKT
KPREEQYNSTYRVVSVLTVLHQDWLNGKEYKCKVSNKALPAPIEKTISKAKGQPREPQ
VYTLPPSRDELTKNQVSLTCLVKGFYPSDIAVEWESNGQPENNYKTTPPVLDSDGSFFLY
SKLTVDKSRWQQGNVFSCSVMHEALHNHYTQKSLSLSPGK

Light Chain of Rituximab (Protein Sequence without app8)

QIVLSQSPAILSASPGEKVTMTCRASSSVSYIHWFQQKPGSSPKPWIYATSNLASGVPVRF
SGSGSGTSYSLTISRVEAEDAATYYCQQWTSNPPTFGGGTKLEIKRTVAAPSVFIFPPSDE
QLKSGTASVVCLLNNFYPREAKVQWKVDNALQSGNSQESVTEQDSKDSTYSLSSTLTLS
KADYEKHKVYACEVTHQGLSSPVTKSFNRGEC

Human IgG1 Fc construct (Protein Sequence without app8)

EPKSCDKTHTCPPCPAPELLGGPSVFLFPPKPKDTLMISRTPEVTCVVVDVSHEDPEVKF
NWYVDGVEVHNAKTKPREEQYNSTYRVVSVLTVLHQDWLNGKEYKCKVSNKALPAPI
EKTISKAKGQPREPQVYTLPPSRDELTKNQVSLTCLVKGFYPSDIAVEWESNGQPENNY
KTTPPVLDSDGSFFLYSKLTVDKSRWQQGNVFSCSVMHEALHNHYTQKSLSLSPGK
